# Improving the depth of data quality or increasing confusion? Reflections on a data analysis involving members of a self‐help group for relatives of people living with dementia

**DOI:** 10.1111/hex.13298

**Published:** 2021-06-08

**Authors:** Antonia Kowe, Stefanie Köhler, Stefan Teipel

**Affiliations:** ^1^ Department ‘Ageing of Individuals and Society’ (AGIS), Interdisciplinary Faculty University of Rostock Rostock Germany; ^2^ Rostock University Medical Center & German Center for Neurodegenerative Diseases (DZNE) Site Rostock/Greiswald Rostock Germany

**Keywords:** caregivers, data analysis, dementia, self‐help groups

## Abstract

**Background:**

Public involvement in research to improve data quality and to empower different stakeholders is good scientific practice, but rarely implemented across all research phases.

**Objective:**

This article reports on an attempt to involve members of a self‐help group for relatives of people living with dementia as co‐researchers in the data analysis in a short‐term format.

**Methods:**

One researcher identified statements about assistive technologies from 17 interviews with people living with dementia and informal caregivers. Two researchers and six co‐researchers independently assigned pre‐defined values to these statements. Subsequently, we compared the values of the researchers and co‐researchers.

**Results:**

The members of the self‐help group identified four original values not considered by the researchers: consent, inclusion, participation and respect.

**Discussion:**

The involvement of co‐researchers led to an improvement in the depth of data quality through the joint identification of values concerning assistive technology. Language barriers between researchers, co‐researchers and interview participants impeded the data analysis.

**Conclusion:**

The challenges and benefits of a participatory data analysis shown here can provide a basis for recommendations for target group‐specific research involvement. Our recommendations relate to the recruitment of co‐researchers, requirements for conducting a participatory data analysis and the participation degree of people involved.

**Patient or Public Contribution:**

The group of co‐researchers participating in the data analysis consisted of relatives of people living with dementia.

## 1 BACKGROUND

Active public involvement in research including lay people as co‐researchers^1^ is an integral part of international scientific efforts.^2^ Patient and public involvement aims to broaden perspectives of all parties involved by incorporating experience‐based knowledge into theory‐driven research.^3^, ^4^, ^5^ For successful research involvement, the continuous participation of such lay co‐researchers in all research phases is recommended.^2^, ^5^, ^6^, ^7^ While public actors are increasingly engaged in the design phase of research, data collection and dissemination of scientific findings,^8^ the public is rarely involved in data analysis.^8^, ^9^, ^10^, ^11^ However, participatory data analysis can increase the depth of data quality.^11^

Public involvement in the data analysis phase of research seems to be particularly difficult compared to other phases^10^ since prior methodological knowledge is crucial to analyse data.^12^ An additional barrier in participatory data analysis is that the data often consist of large amounts of complex material^10^ so that the analysis requires a lot of time^10^, ^13^ and concentration of the co‐researchers in the analysis.^10^ Not all groups that can make a valuable contribution to data analysis have the necessary time and resources to participate in the research phase.

The prerequisites for a participatory data analysis are challenging for any lay person, but are particularly challenging when inviting lay people with cognitive disabilities such as dementia patients and their caregivers with limited time resources to participate. In dementia research, expanding the understanding of researchers through patient and public involvement is essential to find care solutions tailored to the needs of people living with dementia as care recipients and their relatives as caregivers. Evidence from projects involving people living with dementia^12^, ^14^, ^15^, ^16^ and their caregivers^17^ highlights the feasibility of participatory data analysis with these key stakeholders in dementia care. However, there is still a need to extend the existing evidence and resources in terms of structural requirements for patient and public involvement and appropriate recruitment strategies.^17^

The focus of our work was on caregivers of people living with dementia who have a key role in care^18^ and are confronted with specific caregiver burden.^19^, ^20^ Caregivers’ specific perspective may broaden academic researchers’ understanding of dementia. At the same time, caring relatives’ constant obligations to the person living with dementia limit their availability for participation as co‐researchers. In conclusion, there is a need for the involvement of caregivers while considering their specific resources. Here, we aimed to conduct a participatory data analysis with relatives of people living with dementia. We conducted a one‐time, short‐term data analysis session, accommodating the limited resources of the target group. We invited members of a self‐help group for relatives of people living with dementia as our co‐researchers in this study.

Our research question was whether the inclusion of informal caregivers of people living with dementia improves the depth of data quality by enriching the data analysis with additional results or rather complicates the analytical process, leading to confusion. The challenges and opportunities of participatory data analysis are highlighted to provide recommendations.

## 2 THE PROJECT SAMI

The participatory data analysis was carried out within the ongoing study ‘Sensor‐based individualized activity management system for people with dementia in nursing facilities’ (SAMi) with focus on assistive technology. The study aims at the development of a sensor‐based assistance system to support people living with dementia and caregivers in inpatient care. For this purpose, sensor data of people living with dementia were collected with regard to their motion, location and the living context. Furthermore, relevant stakeholders within the dementia care sector were asked about their expectations for a support system and design suggestions. We identified people living with dementia and their caregivers as key stakeholders as people living with dementia will be the direct users of an assistive technology and caregivers often support the person to be cared for in the usage.

Assistive technology can positively impact the life of people living with dementia^21^, ^22^, ^23^, ^24^ and reduce caregiver burden,^23^, ^25^ but the willingness to use technological innovations is relatively low in older people.^24^, ^26^ According to the Technology Acceptance Model (TAM), the willingness to use technological innovations depends on the perceived usefulness and the perceived ease of use.^27^ Early and continuous stakeholder engagement in research to gain knowledge about the needs of future user groups has the potential to increase the acceptance of new technologies by prospective users.^21^, ^23^

In order to develop recommendations for assistive technology, namely a need‐based indoor navigation aid, semi‐structured interviews were conducted with people living with dementia (n = 10) and informal caregivers (n = 10). Data were collected by one researcher (AK) from October 2019 to February 2020. The project and the results are described in detail in another article. We followed the Value Sensitive Design approach, which includes the identification of values of future users for the design of assistive technology.^28^, ^29^ A value is defined as something that has a meaning in the lives of individuals or groups.^28^, ^29^ In terms of assistive technology, values express certain conditions that technology must meet in order to be used.^28^ The technology design approach should be strengthened by a participatory data analysis.

## 3 METHODS

**FIGURE 1:**
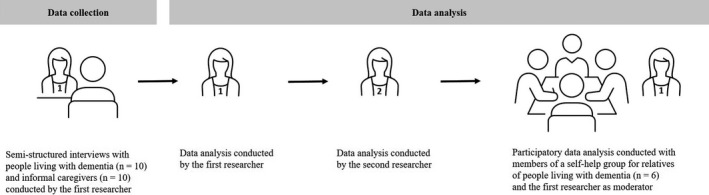
Methods used for data analysis with first researcher (AK), second researcher (SK) and co‐researchers, abbreviations: n‐ number, created with Power‐user

The participatory data analysis was part of a multi‐stage process consisting of data collection by one researcher (AK), a data analysis by two researchers (AK, SK) and a participatory data analysis with the co‐researchers. The methodological approach used here is shown in Figure 1.

### 3.1 Data collection

The data collection was done by one researcher (AK). Interview participants were recruited from the memory clinic of the Rostock University Medical Center and the geriatric ward of a regional hospital. Seventeen interviews with people living with dementia (n = 10) and informal caregivers (n = 10) were conducted. Three interviews were dyadic interviews at the request of people living with dementia. The interviews took place in the rooms of the memory clinic, in the rooms of the hospital and in the private apartments of the interviewees. The semi‐structured questionnaire for the interviews included questions on the current use of assistive technology, barriers to use them and expectations of user‐friendly technologies. The interviews were recorded on tape and written down pseudonymized as smooth verbatim transcripts.

### 3.2 Data analysis

Firstly, one researcher (AK) identified the interview statements related to assistive technology in the transcripts and formed a subset of data only including these statements to reduce the large amount of data to a manageable amount similar to a previous study.^15^ One example of a statement is given here:

‘If it [a technological device] is like a little mobile phone like this, and if you have it like this, that would give him [husband with dementia] safety, I think. He always gets hectic when he has to ask, when he does not know what's going on.’ (caregiver of a person living with dementia)

Secondly, the researcher who conducted the interviews (AK) and another researcher (SK) independently attributed values to the statements related to assistive technology. Data analysis was carried out by two researchers in order to obtain higher reliability. We decided to use an a priori defined list of values (Table 1) to increase the comparability of results between the researchers and co‐researchers as well as to prioritize previously collected values to achieve inter‐rater reliability.^11^ The values were derived by a consensus process between interdisciplinary academic researchers and a literature review related to the core topics selected by the interdisciplinary research group^30^ and can be found in Table 1. Values were divided into values of people living with dementia and values of caregivers, with most values occurring in both groups. The values were attributed to the interview statements.

**TABLE 1:** Values important for people living with dementia and caregivers regarding assistive technology, ordered alphabetically, based on a previous study^30^ (in the study, human rights were also identified as a value, which we did not include for our study), *values only for people living with dementia, **values only for caregivers

Autonomy*	Inclusion	Patient‐caregiver satisfaction	Safety
Consent	Independence	Privacy*	Security
Dignity	Interdependence	Quality of Life	Surveillance**
Emergency help	Mobility**	Relief/respite	Trust
Freedom	Participation	Respect	Well‐being

Thirdly, our co‐researchers conducted the data analysis by assigning values to the statements. There was one member of the self‐help group who was also an interview participant; otherwise, there was no overlap between the interview participant group and the co‐researchers. Here, we followed the recommendation from a previous study^13^ to involve co‐researchers in data analysis who did not participate in the data collection. Access to the self‐help group was provided by directly addressing the group leader as gatekeeper. At the previous group meeting, the group leader had reported on the project and asked the participants for their agreement to participate. The group meets once a month for two hours.

At the beginning of the meeting of the self‐help group, the researcher (AK) acts as moderator of the analysis session and explained the purpose of the study and the method of participatory data analysis. There was no additional training for the participants to keep the effort for the co‐researchers low and the duration of the participatory data analysis short. The participants received pens and handouts with the pseudonymized statements about assistive technology from the interviews, to which they were then to assign the values from Table 1. Each co‐researcher had a handout with six to seven statements. The participants had different statements and were not informed whether these were quotes from people living with dementia or caregivers. The values were listed on the handouts, already sorted by values of people living with dementia and values of caregivers according to the statements on the handouts.

### 3.3 Ethics

Written informed consent was obtained from all participants of the participatory data analysis. For the procedure, an ethical approval was obtained from the Ethics Committee of the Rostock University Medical Center (A 2020‐0064) on 20 March 2020.

## 4 RESULTS

A total of six relatives of people living with dementia from one self‐help group (including the group leader) were involved in the participatory work. The co‐researchers had a mean age of 63 years (range 29‐79 years). Five co‐researchers (83.3%) were female. The analysis session was held on 16 June 2020 in the usual meeting place of the self‐help group. The analysis session took 30 minutes.

The researcher who moderated the analysis session gave a short introduction at the beginning and then received one question from a co‐researcher to the analysis procedure. At the beginning of the individual work, the co‐researchers hesitated and behaved cautiously. The researcher addressed the situation by re‐explaining the task and importance of participatory research.

Grammatical errors impaired the co‐researchers’ understanding of the interviewees’ statements. In addition, the values were not easy to understand for the co‐researchers. One co‐researcher recommended that the co‐researchers should discuss the values prior to the joint data analysis and, if necessary, that synonyms be provided in everyday language. During the analysis, the co‐researchers had the opportunity to ask the moderator questions about the research approach and the values. One of them asked about the definition of the value ‘surveillance’. The initial reluctance of the co‐researchers decreased during the course of the analysis session and the subsequent discussion became informal, with the group leader of the self‐help group supporting the moderator.

The first researcher (AK) identified a total of 31 statements from the interviews with people living with dementia and relatives on assistive technology. The first researcher (AK) assigned a total of 14 values to the statements. The second researcher (SK) also found 14 values regarding assistive technology in the statements. The two researchers identified four values differently. The members of the self‐help group assigned 19 values to the statements. One example for the values identified by the researchers and co‐researchers is shown in Table 2.

**TABLE 2:** Example of an assignment of values to an interview statement by different research group members

Statement	Values identified by the first researcher	Values identified by the second researcher	Values identified by the co‐researchers
*‘*If it is like a little mobile phone like this, and if you have it like this, that would give him safety, I think. He always gets hectic when he has to ask, when he does not know what's going on.’	Safety, independence	Safety, independence	Emergency help, independence, patient‐caregiver satisfaction, quality of life, well‐being

Through the research involvement of the members of the self‐help group, an improvement in depth of data quality by four values was achieved compared to the values identified by the researchers (see Table 3). The values only found by the members of the self‐help group were consent, inclusion, participation and respect.

**TABLE 3:** Values identified by the researchers and the members of the self‐help group for relatives of people living with dementia with the number (n) of times they have been identified in the interview statements

Values identified by the first researcher (n)	Values identified by the second researcher (n)	Values identified by the co‐researchers (n)
Relief/Respite (11)	Safety (12)	Independence (11)
Safety (8)	Relief/Respite (10)	Quality of life (9)
Emergency help (7)	Emergency help (7)	Emergency help (7)
Trust (7)	Independence (6)	Relief/Respite (7)
*Surveillance* (4)	Mobility (5)	**Consent** (6)
Dignity (2)	*Patient‐caregiver satisfaction* (3)	Safety (6)
Independence (2)	Trust (3)	Well‐being (6)
Security (2)	Freedom (2)	Autonomy (5)
Well‐being (2)	Security (2)	Freedom (5)
Autonomy (1)	Well‐Being (2)	Patient‐caregiver satisfaction (4)
Freedom (1)	Autonomy (1)	Trust (4)
Mobility (1)	Dignity (1)	Dignity (3)
Privacy (1)	*Interdependence* (1)	**Inclusion** (3)
*Quality of life* (1)	Privacy (1)	Mobility (3)
Consent (0)	Consent (0)	Surveillance (2)
Inclusion (0)	Inclusion (0)	**Participation** (1)
Interdependence (0)	Participation (0)	Privacy (1)
Participation (0)	Quality of life (0)	**Respect** (1)
Patient‐caregiver satisfaction (0)	Respect (0)	Security (1)
Respect (0)	Surveillance (0)	Interdependence (0)

Note

The values are ranked according to frequency in which they were mentioned. Values that have been identified equally frequently are listed alphabetically. Values that were only found by one of the two researchers are written in italics. Values only identified by the members of the self‐help group are written in bold.

The two researchers hardly differed in the values that they most often attributed to the statements: relief/respite, safety and emergency help. The co‐researchers frequently assigned the value of ‘independence’, which was identified by the researchers much less often. 'Quality of life' as the second most frequently identified value among the members of the self‐help group was only found once by the first researcher and not at all by the second researcher. 'Emergency help' was also the third most frequently identified value by the members of the self‐help group.

## 5 DISCUSSION

We conducted a participatory analysis of qualitative data with members of a self‐help group for relatives of people living with dementia. Our research approach allowed us to invite six relatives of people living with dementia as a key stakeholder group in dementia care and involve the caregivers as co‐researchers while accommodating their limited time resources. This participatory data analysis enabled an improvement in the depth of data quality by four values.

### 5.1 Challenges and opportunities of participatory data analysis

Through the research involvement of the lay people, four values from the interview statements of people living with dementia and relatives could be identified. This finding of an improvement in the depth of data quality through participatory data analysis is congruent with previous studies including people living with dementia^12^, ^15^, ^16^ and caregivers.^17^ The values only found by our co‐researchers were consent, inclusion, participation and respect. These values were related to assistive technology, but can also be generalized. They highlighted the importance of public involvement to include people directly and indirectly affected by a condition in a respectful way in science. The participatory data analysis showed how different the perspectives of caregivers and researchers are and that inclusion is therefore urgently needed to understand the ‘real world’ of those involved.^31^, ^32^ In addition, the question arises why the researchers did not identify these core values in dementia research, in particular ‘consent’, which was identified with a high frequency among the co‐researchers. A subsequent discussion after the data analysis session on the definition of the values and the rationale for the assignment of the values to the statements could have provided decisive clues for the different results among the researchers and the co‐researchers. We tried to limit the length of the data analysis session due to the time limits and daily obligations of caregivers. However, a one‐time session did not provide the necessary depth of analysis and a 30‐minute session was not sufficient to perform participatory data analysis comprehensively. It is advisable to hold several sessions and to divide them into different thematic sections, such as the introduction to the research method, the data analysis, the subsequent discussion of the results and the evaluation of the method.

Training of co‐researchers on a pre‐project basis may be difficult due to their time constraints. Establishing a board of trained co‐researchers beyond any single project might address this issue. This would deepen stakeholders’ research involvement by inviting their needs and perspectives. However, such a board would require adequate compensation of co‐researchers’ efforts and institutional infrastructure to be sustainable in the long term. Non‐monetary forms of remuneration for research involvement must also be considered in order to enable cooperation between researchers and co‐researchers on an equal footing.^33^ This was beyond the current scope of the project but deserves further discussion.

The chosen method of data analysis with a list of a priori defined values enabled a structured assignment of values and comparability between the results of the researchers and co‐researchers. At the same time, the co‐researchers were limited by the pre‐defined list of values and could not add any values. The method of inter‐rater reliability is criticized for lack of depth in the analysis of complex data.^11^ With regard to the exploratory nature of qualitative research, it seems advisable to give co‐researchers the opportunity to add values and discuss them afterwards.

The self‐help group leader's encouragement of the discussion promoted an informal atmosphere. This underlines the previously established importance of a mediator between those involved in research.^34^, ^35^

We expected that caring relatives, as experts on the living environment of those affected, would interpret the linguistic characteristics of people living with dementia with ease. Unexpectedly, this was where the biggest problems appeared. The problems of understanding people living with dementia other than their personal relative with dementia emphasized that people affected by the condition are a heterogeneous group^36^ and informal caregivers often only know the individual symptoms and characteristics of their relative with dementia. It would be valuable to investigate whether formal caregivers, who professionally care for many people living with dementia, are more likely to know the linguistic anomalies and thus be able to enrich the data analysis with their professional care experience. An important challenge was the communication gap between researchers and co‐researchers. For the co‐researchers, the scientific terminology of the values was difficult to understand. This may be one reason for the reservations of the co‐researchers at the beginning of the analysis session. On the other hand, it was difficult for the researcher to present the analysis method and the project in an easy‐to‐understand way. Awareness in research concerning the relationship of public involvement and effective science communication is increasing.^37^

### 5.2 Participation degree

As criticized in previous studies,^12^, ^38^ the decision‐making power in our study remains with the academic researchers. Academic researchers selected the statements provided to the co‐researchers, selected the values, moderated the analysis session and led the discussion. Future investigations should aim on strategies to include co‐researchers in a greater scope with appropriate effort for the participants.

All in all, we could only achieve a participation degree of consultation in which the power in the research process remains with the researchers.^39^ Tuffrey‐Wijne and Butler^38^ concluded that despite the power discrepancy between researchers and co‐researchers in their study, the co‐researchers contributed valuable input into the research team's decision‐making. Based on their^38^ and our experiences, it can be deduced that researchers should have realistic expectations on participatory research projects and that co‐researchers should be able to participate as much as possible within the scope of the project possibilities: Research involvement must be person‐centred, which means that co‐researchers can decide for themselves whether, when and how they want to be involved in research. This person‐centred approach in research involvement could reduce the risk that researchers will consider projects with only a low level of participation to be participatory and counteracts tokenism, because it creates a bottom‐up principle in determining the level of participation by co‐researchers. Furthermore, this approach follows the recommendation to consider the needs, preferences and resources of persons involved in research.^2^, ^5^, ^6^, ^38^, ^40^

### 5.3 Key learning points

- Participatory data analysis with informal caregivers of people living with dementia enables greater depth of the analysis and improves data quality.
- Participatory data analysis was kept short due to the limited time resources of the co‐researchers. However, it became apparent that several sessions would have been necessary to prepare the co‐researchers adequately for the analysis and to evaluate the results together afterwards.
- The level of participation must be based on the preferences and abilities of the co‐researchers. In line with the approach of person‐centredness, co‐researchers should determine their level of decision‐making power in projects.

### 5.4 Limitations

We did not discuss the values prior to the joint data analysis with the co‐researchers. Questions about the meaning of a value from co‐researchers could be answered individually, but it remains unclear whether all co‐researchers understood all values. Furthermore, co‐researchers were only confronted with a small set of data. The data basis for the given recommendations is still limited and needs to be expanded by future research.

A member of the self‐help group was also an interviewee. It should be noted that self‐help groups do not serve the purpose of research and that members should act as co‐researchers in a setting different to group meetings when long‐term involvement in research is planned. A self‐help group may be understood as an opportunity to access the target group but does not replace a research advisory board.

## 6 CONCLUSION

In summary, there has been an improvement in the depth of data quality through participatory data analysis. The challenges of implementing the participatory approach inform the design of public participation in the analysis research phase. We derived recommendations for the recruitment of co‐researchers, time requirements for conducting a participatory data analysis, the need for a mediator and the necessity of the determination of the participation degree by the co‐researchers.

## CONFLICT OF INTEREST

Antonia Kowe is fellow of the program ‘Freies Wissen’ by Wikimedia Deutschland. Stefan Teipel has done the listed works below (all in Germany). MSD Sharp & Dohme GmbH, Lindenplatz 1, 85540 Haar: 11/09/2018 – Quality circle for physicians in Kühlungsborn, Talk: ‘Dementia and Diabetes – current report’. 14/11/2018 – MSD Expert‐forum: NAB Alzheimer in Munich, participator as consultant. 13/08/2019 –Event ‘Diabetes and Dementia’ in Rostock, Talk: ‘Dementia and Diabetes – current report’. ROCHE Pharma AG, Emil‐Barell‐Str. 1, 79639 Grenzach‐Wyhlen: 12/09/2019 ‐ 3. Nationales Advisory‐Board in Frankfurt (Main), participator as consultant. 27/09/2019 – ROCHE Symposium at the DGN Congress in Stuttgart, Talk: ‘Amyloid as target for diagnosis and treatment in Alzheimer´s disease’. Biogen GmbH, Riedenburger Straße 7, 81677 München: 23/04/2020 – Biogen Advisory Board Session. 28/04/2020 – Biogen Advisory Board Session. 18/09/2020 – Biogen Advisory Board Session. Dr Willmar Schwabe GmbH & Co. KG, Ottostr. 24, 76227 Karlsruhe: 22/01/2021 – Virtual Advisory Board. The other author declares that there is no conflict of interest.

## Data Availability

Data are available on request due to privacy/ethical restrictions.
